# Penehyclidine combined with antiemetics for preventing postoperative nausea and vomiting: A meta-analysis of randomized control trials and trial sequential analysis

**DOI:** 10.1097/MD.0000000000042908

**Published:** 2025-06-20

**Authors:** Hongwei Zhang, Jianqiao Zheng, Lu Zhang, Li Du

**Affiliations:** a Department of Anesthesiology, Sichuan Clinical Research Center for Cancer, Sichuan Cancer Hospital & Institute, Sichuan Cancer Center, University of Electronic Science and Technology of China, Chengdu, China; b Department of Anesthesiology, West China Hospital, Sichuan University, Chengdu, Sichuan, China.

**Keywords:** meta-analysis, penehyclidine, PONV, postoperative nausea and vomiting

## Abstract

**Background:**

Postoperative nausea and vomiting (PONV) is one of the most common adverse complications associated with anesthesia and after surgical procedures. PONV is related to patient dissatisfaction and can lead to several postoperative complications. This study aimed to examine the efficacy and safety of penehyclidine combined with antiemetics in preventing PONV.

**Methods:**

We searched English databases (including PubMed, Web of Science, Ovid Medline, Embase and Cochrane Library), Chinese electronic databases (including China National Knowledge Infrastructure, Wanfang, VIP, and Chinese Biomedical Literature) and trial registry databases to find randomized controlled trials researching the clinical validity of penehyclidine combined with antiemetics for PONV. The retrieval time was up to January 2025, without publication date restrictions. Articles published in the English and Chinese languages were considered. The primary outcome was the incidence of PONV within and over 24 hours postoperatively. The secondary outcomes were the incidence of different severities of PONV, incidence of postoperative rescue antiemetic therapy and postoperative complications. Quality assessment was conducted with the Cochrane Collaboration’s risk of bias tool and grading of recommendations assessment, development and evaluation method. Subgroup analyses were performed according to the postoperative observation period. Trial sequential analysis was performed to validate the reliability of the primary outcome. Publication bias was assessed by funnel plots and Egger’s regression test.

**Results:**

Sixteen randomized controlled trials comprising 1561 participants were included. Compared with the control group, penehyclidine combined with antiemetics provided a lower incidence of PONV (risk ratio [RR]: 0.65; 95% CI: 0.57–0.74; *P* < .00001; *I*^2^ = 11%; low quality), lower incidence of severe PONV over 24 hours postoperatively (RR: 0.29; 95% CI: 0.15–0.56; *P* = .0003; *I*^2^ = 0%; low quality), and a lower incidence of postoperative rescue antiemetic therapy (RR = 0.42, 95% CI: 0.26–0.69; *P* = .0006; *I*^2^ = 58%; low quality). However, it was associated with a higher incidence of dry mouth (RR: 3.10, 95% CI: 2.28–4.20; *P* < .00001; *I*^2^ = 19%; low quality), but did not increase the incidence of other anticholinergic-related complications (RR: 1.08; 95% CI: 0.91–1.28; *P* = .38; *I*^2^ = 2%; low quality).

**Conclusion:**

Compared with antiemetics, penehyclidine combined with antiemetics could provide better prevention efficiency for PONV, with lower incidence of PONV, lower incidence of severe PONV over 24 hours postoperatively and lower incidence of postoperative rescue antiemetic therapy.

## 1. Introduction

Postoperative nausea and vomiting (PONV) defined as any nausea, and vomiting within the first 24 to 48 hours after surgery in inpatients, is one of the most common adverse complications associated with anesthesia and surgical procedures, and is strongly related to patient dissatisfaction.^[1–3]^ PONV is a highly distressing experience and is associated with significant patient dissatisfaction.^[4,5]^ Compared with pain and decreased mental alertness, PONV is reported to be the most undesirable condition in postoperative recovery.^[6]^ In addition, PONV can lead to several postoperative complications, including water-electrolyte imbalance, surgical wound dehiscence, esophageal tear, increased intracranial pressure, and pulmonary aspiration.^[7–9]^ Moreover, PONV can lead to a longer length of postanesthesia care unit,^[10]^ delayed discharge,^[11]^ unplanned hospital admission,^[12]^ and increased overall health care costs.^[13]^ Unfortunately, PONV is estimated to affect 30% of all patients and up to 80% of high-risk patients.^[14,15]^ Even in patients receiving antiemetics, the reported incidence of PONV still ranges from 18% to 38%.^[16–19]^ Therefore, reducing the incidence of PONV is clinically important.

Combination therapy involving 2 or more antiemetics that act on different receptors for the prevention of PONV has been recommended by the Consensus Guidelines for the Management of PONV as a risk-based approach to PONV prophylaxis.^[14]^ For patients with 1 or 2 risk factors for PONV, a combination of 2 antiemetic agents is recommended, and for patients with 3 or more risk factors, at least 3 antiemetic agents are recommended.^[14]^ The use of combination therapy for the prevention of PONV has since been firmly established in current anesthesia practices. Currently, the common pharmacological options for the management of PONV include 5-hydroxytryptamine receptor antagonists, corticosteroids, neurokinin-1 receptor antagonists, antihistamines, dopamine receptor antagonists, and anticholinergics.^[14]^ However, these antiemetic drugs remain unsatisfactory, and are associated with various adverse effects, including headache, sedation, constipation, extrapyramidal symptoms, arrhythmia and QT prolongation, hyperglycemia, immunosuppression and poor wound healing, dry mouth, urinary difficulties, dizziness, and visual disturbances.^[20–23]^

In recent years, progress has been made in the combination with antiemetic drugs, and anticholinergics have been used to reduce the incidence of PONV.^[4,5]^ Anticholinergics are widely used as premedication to reduce salivary secretion, even anticholinergics have antiemetic effects but are not commonly used as perioperative antiemetic drugs. Penehyclidine is a new anticholinergic agent with a long elimination half-life and highly selective antagonistic action for type 3 and type 1 muscarinic acetylcholine receptors.^[24]^ Penehyclidine has a lower incidence of adverse effects than do other anticholinergics and is a commonly used premedication to reduce salivary secretion.^[25,26]^ Type 1 muscarinic acetylcholine receptors are expressed at high levels in the vestibular system, and the type 3 muscarinic acetylcholine receptors play important roles in the pathogenesis of PONV.^[22,27,28]^ Considering that muscarinic acetylcholine receptors are involved in the development of PONV through multiple mechanisms, penehyclidine may play a role in preventing PONV.

Recent clinical studies have shown that penehyclidine for the prevention of PONV has made good progress in some surgical procedures, such as ophthalmology, laparoscopic bariatric surgery, gynecological laparoscopic surgery, thyroid surgery and stomatology surgery.^[29–34]^ However, it has also been reported that penehyclidine does not decrease the incidence or the severity of PONV in patients undergoing laparoscopic bariatric surgery.^[35]^ As a premedication to reduce salivary secretion, the clinical effectiveness of penehyclidine combined with other antiemetics for the prevention of PONV remains inconsistent and unclear on the basis of the available clinical studies. Therefore, we conducted a meta-analysis with TSA to assess the efficacy of penehyclidine combined with antiemetics in preventing PONV.

## 2. Materials and methods

### 2.1. Protocol and registration

This study was conducted in accordance with the Preferred Reporting Items for Systematic Reviews and Meta-Analyses guidelines, and the Cochrane Handbook for Systematic Review of Interventions.^[36,37]^ The review protocol was registered with the International Prospective Register of Systematic Reviews (PROSPERO-CRD42024561090).

### 2.2. Search strategy

The PubMed, Web of Science, Cochrane Library, Ovid Medline, Embase and Chinese databases (including the China National Knowledge Infrastructure, Wanfang database, VIP databases and Chinese Biomedical Literature) were searched from inception to January 2025 to identify randomized controlled studies of participants who received penehyclidine combined with antiemetics for PONV. The search keywords were as follows: (penehyclidine) AND ([postoperative nausea and vomiting] OR [PONV]). The search terms were translated into Chinese for study identification in Chinese databases. In addition, the list of references, relevant conference literature, and trial registry databases (WHO International Clinical Trials Registry Platform, Clinical Trials.gov, and Chinese Clinical Trials Registry) were scrutinized to identify additional studies. The preliminary search strategy and the details of the search process used for each database are given in Appendix 1, Supplemental Digital Content, https://links.lww.com/MD/P216. Articles published in the English and Chinese languages were considered. The references of related reviews and included studies were manually searched to obtain more appropriate relevant studies.

### 2.3. Inclusion and exclusion criteria

Studies were selected via the PICOS strategy (participants, interventions, comparators, outcomes, and study designs).

The inclusion criteria for the studies included in this meta-analysis were as follows:

1. Participants: Patients who underwent surgery under general anesthesia;
2. Types of interventions: Penehyclidine combined with different antiemetic drugs used for PONV (Penehyclidine + Antiemetics group);
3. Types of comparators: Antiemetic drugs other than penehyclidine for PONV (Antiemetics group);
4. Types of outcomes:
  ➢ The primary outcome:
  The incidence of PONV within and over 24 hours postoperatively.
  PONV was defined as follows:
    (1) The development of any nausea, retching, or vomiting after surgery.^[33]^
    (2) At least 1 episode of nausea or vomiting or a combination of both symptoms.^[34]^
    (3) At least 1 episode of nausea, vomiting, retching, or any combination of these symptoms.^[35]^
  ➢ The secondary outcomes were as follows:
    (1) Incidence of different severities of PONV: defined as the ratio of the number of patients who evaluated to the corresponding severity of PONV.
    The severity of PONV was evaluated as follows:
      (1) Mild PONV: occurrence of mild nausea or 1 episode of vomiting^[30,34]^; nausea but no vomiting^[35,38,39]^; mild nausea, abdominal discomfort, but no vomiting^[40]^;
      (2) Moderate PONV: moderate-to-severe nausea, or vomiting for 2 times or more, or any nausea that required only one rescue antiemetic therapy^[30,34]^; mild to moderate vomiting^[35,38]^; 1 to 2 episodes of vomiting within 30 minutes^[39]^; obvious vomiting, no gastric contents vomiting out^[40]^;
      (3) Severe PONV: more than 2 emetic episodes or necessitating more than 1 dose of rescue antiemetics^[30,34]^; severe and frequent vomiting more than 5 times within 24 hours^[35,38]^; more than 3 episodes of vomiting within 30 minutes^[39]^; severe vomiting, vomiting of gastric juice and other contents, and needing drugs.^[40]^
    2) Incidence of postoperative rescue antiemetic therapy: defined as the ratio of the number of patients who had received antiemetics (regardless of how many times the patient had received it) divided by the total number of patients.^[29]^
    3) Incidence of postoperative complications: included dry mouth and other anticholinergic-related complications (including headache; dizziness; fever, defined as a temperature >37.5°C; and urinary retention, defined as required urine recatheterization).^[33]^
  ➢ Exploratory outcomes
    (1) Incidence of postoperative nausea (PON): PON is defined as a subjective sensation of an unpleasant feeling associated with an awareness of wanting to vomit.^[29]^
    (2) Incidence of postoperative vomiting (POV): POV is defined as the forceful expulsion of gastric contents through the mouth, or dry-retching, and was assessed by the patient’s response of yes or no to questioning.^[29]^ Vomiting was diagnosed when patients retched or expelled intragastric contents.^[33]^
5. Types of studies: Randomized controlled trials (RCTs) published in English or Chinese were included. Studies with the following situations were excluded: studies that only compared the effectiveness of monotherapy with penehyclidine for PONV; studies without randomization for treatment; studies whose data could not be extracted for statistical analysis; and duplicate publications, editorials, letters, abstracts from conferences, reviews, and ongoing trials.

### 2.4. Study selection and data extraction

Two authors (Hongwei Zhang and Jianqiao Zheng) were required to screen the retrieved studies independently. Briefly, all titles and abstracts identified in the search were carefully examined, duplicate studies and those that did not match the inclusion criteria were excluded. The same 2 authors (Hongwei Zhang and Jianqiao Zheng) read the full text of each remaining study to select any potentially relevant articles. Any disagreements were resolved through discussion and the involvement of a third reviewer (Lu Zhang). A fourth author (Li Du) checked all procedures before approving the data extraction.

Two authors (Hongwei Zhang and Jianqiao Zheng) independently extracted data from the included studies following a data acquisition Excel form (Excel version 2013, Microsoft Inc., Washington). The form included the first author’s name, publication year, participants’ demographic data (age, gender, BMI, American Society of Anesthesiologist classification), sample size, detailed information on general anesthesia management (including anesthesia induction and maintenance), detailed information on the prevention of PONV (including type of antiemetics, administration time and dose of penehyclidine and antiemetics) and outcomes. A third reviewer (Li Du) was required to double-check the data to ensure consistency. We attempted to contact the corresponding author by email to obtain the original data when the information and data were missing or incomplete in any study. Useful numerical data in the graphs were extrapolated by Adobe Photoshop, if necessary and feasible.^[41]^ The study characteristics and demographic data are summarized in Table 1.

**Table 1:** The characteristics of the included studies.

Study	Year	Participants and surgery	General anesthesia protocols	Group	Intervention information	Sample	Age, yr (MD ± SD)	Gender (M/F)	BMI (kg/m^2^)	ASA classification (1/2/3/4)	Outcomes
Zhang, et al^[29]^	2012	Patients were scheduled for gynecological laparoscopic surgery	Anesthesia induction: Routine protocol	Penehyclidine + Antiemetics	Penehyclidine (10 μg/kg im, MTD: 1 mg) before anesthesia induction + tropisetron (0.1 mg/kg: 10 mL, iv; MTD: 5 mg) after the surgery	40	30 ± 8	0/40	NA	NA	③④⑥
			Anesthesia maintenance: TIVA	Antiemetics	Atropine (10 μg/kg im, MTD: 0.5 mg) before anesthesia induction + tropisetron (0.1 mg/kg: 10 mL, iv; MTD: 5 mg) after the surgery	40	33 ± 7	0/40	NA	NA	
Zhang, et al^[31]^	2011	Patients (ASA I–II) scheduled for gynecological laparoscopic surgery	Anesthesia induction: Routine protocol	Penehyclidine + Antiemetics	Penehyclidine (10 μg/kg: 2 mL, iv) before anesthesia induction + Tropisetron (0.06 mg/kg: 20 mL, iv) at the end of the surgery	20	NA	NA	NA	NA	⑥
			Anesthesia maintenance: TIVA	Antiemetics	Saline (2 mL iv) before anesthesia induction + Tropisetron (0.06 mg/kg: 20 mL, iv) at the end of the surgery	20	NA	NA	NA	NA	
Wang, et al^[33]^	2022	Adult patients (18–60 yr, 18 kg/m^2^ ≤ BMI < 30 kg/m^2^) scheduled for bimaxillary orthognathic surgery	Anesthesia induction: Routine protocol + dexamethasone (10 mg)	Penehyclidine + Antiemetics	Penehyclidine (0.5 mg: 5 mL, iv before anesthesia induction) + Tropisetron (2 mg iv at the end of the surgery + 10 mg: PCIA)	117	24 (21, 28)	35/82	20.3 (18.8, 22.0)	97/20/0/0	①②③④
			Anesthesia maintenance: CIVIA	Antiemetics	Saline (5 mL iv) before anesthesia induction + Tropisetron (2 mg iv at the end of the surgery + 10 mg: PCIA)	118	25 (22, 28)	37/81	20.0 (18.8, 22.7)	87/31/0/0	
Zhao, et al^[34]^	2024	Women (ASA II–III, >18 yr old) scheduled for gynecological laparoscopic surgery	Anesthesia induction: Routine protocol + dexamethasone	Penehyclidine + Antiemetics	Penehyclidine (10 μg/kg iv during anesthesia induction + PCIA: 10 μg/kg, MTD: 0.5 mg) + ondansetron (8 mg: PCIA)	46	44.1 ± 11.0	0/46	23.8 ± 4.1	0/28/18/0	①②③④⑤⑥
			Anesthesia maintenance: CIVIA	Antiemetics	Saline (volume-matched 0.9% saline iv during anesthesia induction) + ondansetron (8 mg: PCIA)	46	48.8 ± 12.0	0/46	24.9 ± 4.8	0/27/19/0	
Ding, et al^[35]^	2023	Patients (ASA I–III, 18–60 yr) scheduled for LBS under general anesthesia	Anesthesia induction: Routine protocol + dexamethasone (10 mg)	Penehyclidine + Antiemetics	Penehyclidine (0.5 mg iv) + Palonosetron (0.25 mg iv) 30 min before the end of surgery	221	33 ± 8	68/153	38 ± 7	0/103/118/0	①②④⑤⑥
			Anesthesia maintenance: TIVA	Antiemetics	Saline (same volume-matched iv) + Palonosetron (0.25 mg, iv) 30 min before the end of surgery	113	34 ± 9	25/88	38 ± 7	0/49/64/0	
Liu^[38]^	2016	Patients (ASA I–II) scheduled for laparoscopic surgery	Anesthesia induction: Routine protocol	Penehyclidine + Antiemetics	Penehyclidine (0.5 mg iv) + tropisetron (4 mg: 2 mL, iv) 30 min before the end of surgery	30	44.0 ± 9.0	NA	NA	NA	①②④
			Anesthesia maintenance: CIVIA	Antiemetics	Tropisetron (4 mg: 2 mL, iv) 30 min before the end of surgery	30	44.0 ± 8.7	NA	NA	NA	
Si, et al^[39]^	2008	Patients (ASA I–II) scheduled for middle ear surgery	Anesthesia induction: Routine protocol	Penehyclidine + Antiemetics	Penehyclidine (1 mg iv) + tropisetron (2 mg: 10 mL, iv) before anesthesia induction	20	34.4 ± 7.5	10/10	NA	NA	①②
			Anesthesia maintenance: CIVIA	Antiemetics	Tropisetron (2 mg: 10 mL, iv) before anesthesia induction	20	35.8 ± 8.6	7/13	NA	NA	
Chen, et al^[40]^	2014	Patients (ASA I–II) scheduled for LC	Anesthesia induction: Routine protocol	Penehyclidine + Antiemetics	Penehyclidine (0.5 mg iv) + Azasetron (10 mg iv) at the beginning of the surgery	40	NA	NA	NA	NA	①②
			Anesthesia maintenance: TIVA	Antiemetics	Azasetron (10 mg iv) at the beginning of the surgery	40	NA	NA	NA	NA	
Zhong^[42]^	2021	Patients (ASA I–II) scheduled for thyroid cancer surgery	Anesthesia induction: Routine protocol	Penehyclidine + Antiemetics	Penehyclidine (0.5 mg iv) + Dolasetron (12.5 mg iv) before anesthesia induction	30	61.25 ± 1.54	18/12	NA	NA	①④
			Anesthesia maintenance: TIVA	Antiemetics	Dolasetron (12.5 mg iv) before anesthesia induction	30	60.91 ± 1.45	16/14	NA	NA	
Chen^[43]^	2017	Patients scheduled for gynecological laparoscopic surgery	Anesthesia induction: Routine protocol.	Penehyclidine + Antiemetics	Penehyclidine (10 μg/kg iv, MTD: 1 mg) + Tropisetron (0.1 mg/kg iv, MTD: 5 mg) 30 min before the end of surgery	50	NA	NA	NA	NA	①③
			Anesthesia maintenance: TIVA	Control	Tropisetron (0.1 mg/kg iv, MTD: 5 mg) 30 min before the end of surgery	50	NA	NA	NA	NA	
Du^[44]^	2014	Patients (ASA I–II) scheduled for LC	Anesthesia induction: Routine protocol	Penehyclidine + Antiemetics	Penehyclidine (10 μg/kg: 5 mL, iv) before anesthesia induction + tropisetron (5 mg iv) at the end of surgery	20	NA	NA	NA	NA	⑥
			Anesthesia maintenance: CIVIA	Antiemetics	Saline (5 mL iv) before anesthesia induction + tropisetron (5 mg iv) at the end of surgery	20	NA	NA	NA	NA	
Wang, et al^[45]^	2013	Patients (ASA I–II) scheduled for gynecological laparoscopic surgery	Anesthesia induction: Routine protocol	Penehyclidine + Antiemetics	Penehyclidine (10 μg/kg:5 ml, iv) before anesthesia induction + tropisetron (5 mg: 5 ml, iv) after the surgery	40	45 ± 16	NA	NA	NA	⑥
			Anesthesia maintenance: CIVIA	Antiemetics	Saline (5 mL iv) + tropisetron (5 mg: 5 mL, iv) after the surgery	40	43 ± 14	NA	NA	NA	
Zheng, et al^[46]^	2011	Patients scheduled for abdominal surgery under general anesthesia	Anesthesia induction: Routine protocol	Penehyclidine + Antiemetics	Penehyclidine (10 μg/kg: 5 mL, iv) before anesthesia induction + tropisetron (5 mg iv) the end of the surgery	20	NA	NA	NA	NA	①
			Anesthesia maintenance: CIVIA	Antiemetics	Saline (5 mL iv) before anesthesia induction + tropisetron (5 mg iv) at the end of the surgery	20	NA	NA	NA	NA	
Yi^[47]^	2009	Patients (ASA I–II) scheduled for LC	Anesthesia induction: Routine protocol	Penehyclidine + Antiemetics	Penehyclidine (0.5 mg: 5 mL, iv) before anesthesia induction + granisetron (3 mg iv) after the gallbladder removed	60	NA	NA	NA	NA	①
			Anesthesia maintenance: TIVA	Antiemetics	Granisetron (3 mg iv) after the gallbladder removed	60	NA	NA	NA	NA	
Ma, et al^[48]^	2008	Patients (ASA I–II) scheduled for abdominal surgery under general anesthesia	Anesthesia induction: Routine protocol	Penehyclidine + Antiemetics	Penehyclidine (10 μg/kg: 5 mL, iv) before anesthesia induction + tropisetron (5 mg: 5 mL, iv) after the surgery	40	NA	NA	NA	NA	①③
			Anesthesia maintenance: CIVIA	Antiemetics	Saline (5 mL iv) before anesthesia induction + tropisetron (5 mg: 5 mL, iv) after the surgery	40	NA	NA	NA	NA	
Dong, et al^[49]^	2007	Patients (ASA I–II) scheduled for LC	Anesthesia induction: Routine protocol	Penehyclidine + Antiemetics	Penehyclidine (10 μg/kg: 5 mL, iv) before anesthesia induction + tropisetron (5 mg iv) at the end of the surgery	40	NA	NA	NA	NA	①③
			Anesthesia maintenance: CIVIA	Antiemetics	Saline (5 mL iv) before anesthesia induction + tropisetron (5 mg iv) at the end of the surgery	40	NA	NA	NA	NA	

Anesthesia induction routine protocol: defined as anesthesia induction was performed by a combination of sedatives (including midazolam, propofol, etomidate), opioid (including fentanyl, sufentanil, remifentanil), and muscle relaxants (including vecuronium bromide, atracurium, atracurium, cis-atracurium, rocuronium bromide).

Outcome description: ① incidence of the PONV; ② Severity of PONV; ③ Incidence of complications; ④ Incidence of postoperative rescue antiemetic therapy; ⑤ Incidence of PON; ⑥ Incidence of POV.

ASA = American Society of Anesthesiologist, BMI = body mass index, CIVIA = combined intravenous-inhalation anesthesia, F = Female, iv = intravenous injection; im: intramuscular injection, LBS = laparoscopic bariatric surgery, LC = laparoscopic cholecystectomy, M = male, MD±SD = mean difference, MTD = maximal total dose, NA = not applicable, PCIA = patient-controlled intravenous analgesia, SD = standard deviation, TIVA = total intravenous anesthesia, yr = year.

### 2.5. Quality evaluation

The risk of bias in the selected studies was independently evaluated by 2 authors (Hongwei Zhang and Lu Zhang) via Cochrane’s risk of bias assessment tool.^[50]^ The methodology included random sequence generation, allocation concealment, blinding of participants and physicians, blinding of outcome assessors, incomplete outcome data, selective reporting, and other biases.^[50]^ The risk of bias components were divided into 3 levels: low risk, unclear risk, and high risk.^[50,51]^ Disagreements were resolved through arbitration by a third reviewer (Li Du).

The Grading of Recommendations Assessment, Development, and Evaluation (GRADE) methodology was used to assess the quality of the evidence for each outcome.^[52]^ The quality of effect estimates was classified as high, moderate, low or very low depending on the risk of bias, consistency, directness, precision, publication bias, large effect, and plausible confounding.^[52]^ The possible results for each category were “no serious limitations” (no downgrading), “serious limitations” (downgraded by 1 level), or “very serious limitations” (downgraded by 2 levels). After the assessment, a table concerning the GRADE evidence profile was created via GRADE profiler version 3.6.1 to rate the quality of all the outcomes.

### 2.6. Statistical analysis

All the outcomes were reported as dichotomous outcome data, and relative risk (RR) with 95% confidence intervals (CIs) were used. Heterogeneity analyses were performed with Review Manager version 5.4 (RevMan, Cochrane Collaboration, Oxford, UK). Statistical heterogeneity was assessed by the standard *χ*^2^ test (α = 0.1) and *І*^2^ test. If *P* ≥ .1 and if *І*^2^ ≤ 50%, the fixed effects model was used. If *P* < .1 or *І*^2^ > 50%, the random-effects model was applied.^[37]^ If heterogeneity was significant, we omitted the included studies one by one from the pooled analyses in turn to find potential sources of heterogeneity. A *P* value <.05 was assumed to indicate statistical significance.

PONV is a common adverse event that emerges within 24 hours after surgery and anesthesia.^[14,53,54]^ We subsequently performed subgroup analyses according to the postoperative observation period, which was categorized as within 24 hours postoperatively and over 24 hours postoperatively. In addition, subgroup analysis was also performed on different types of complications (including dry mouth and other anticholinergic-related complications, such as headache, dizziness, fever, and urinary retention) and different severities of PONV (including mild, moderate, and severe). *P* < .05 was considered statistically significant.

For the primary outcome, publication bias was assessed by visual judgment of funnel plot asymmetry and, more objectively, through Egger’s regression test.^[55,56]^ A level of *P* < .05 was considered statistically significant and indicated potential publication bias. When significant publication bias existed, trim-and-fill analysis was performed to estimate the number of missing studies that might exist.^[57]^ Then, we examined the reduced and adjusted publication bias results of the trim-and-fill analysis, and assessed whether these results were stable.

The TSA is a statistical method used to evaluate the reliability and conclusiveness of cumulative meta-analysis of RCTs.^[58]^ TSA adjusts for the risks of random errors (type I and type II errors) and repetitive testing, aiming to determine if available evidence is sufficient for firm conclusions.^[59]^ For the primary outcome, trial sequential analysis (TSA) was performed. The required information size (RIS) and TSA monitoring boundaries were quantified. For dichotomous outcomes, the RIS was calculated on the basis of the proportion of participants with an outcome in the control group and a relative risk reduction of 30%. The risk of type I error was maintained at 2.5% with a power of 90%. TSA was performed via the TSA program V.0.9.5.10 Beta (http://www.ctu.dk/tsa).^[60]^

## 3. Results

### 3.1. Study retrieval and selection

**Figure 1.**
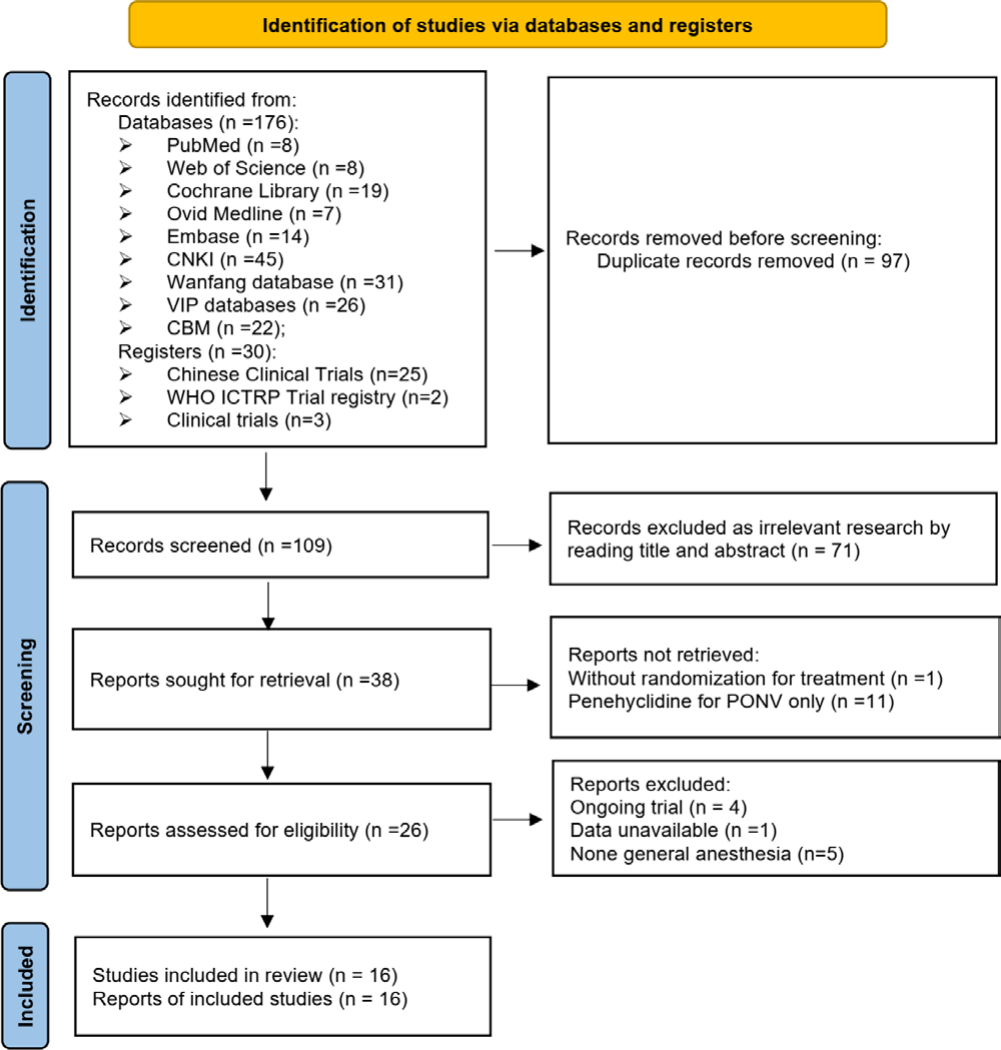
PRISMA flow diagram. PRISMA = Preferred Reporting Items for Systematic Reviews and Meta-Analyses. Source from Page et al.^[36]^ For more information, visit: https://www.prisma-statement.org/prisma-2020.

Our search strategy results are summarized in Figure 1. A total of 176 articles were identified via the database search and 30 records were included in the clinical register databases on penehyclidine for PONV. After removing duplicates (a total of 97 articles and clinical registries) and articles excluded after screening the titles and abstracts revealed that they were unrelated to the study question (n = 71), 38 articles were screened. Twenty two studies were excluded because they failed to meet the predetermined inclusion criteria, and 16 articles involving 1561 patients were included in the final analysis.^[29,31,33–35,38–40,42–49]^

### 3.2. Study characteristics

The characteristics of the included studies are summarized in Table 1. Sixteen RCTs with 1561 participants (Penehyclidine + Antiemetics group: 834 participants; Antiemetics group: 727) were included in the meta-analysis.^[29,31,33–35,38–40,42–49]^ Among the 16 studies, 11 studies involved laparoscopic surgery (including gynecological laparoscopic surgery,^[29,31,34,43,45]^ laparoscopic bariatric surgery,^[35]^ laparoscopic cholecystectomy,^[40,44,47,49]^ general laparoscopic surgery^[38]^), 2 studies involved abdominal surgery,^[46,48]^ and 1 study each involved bimaxillary orthognathic surgery,^[33]^ thyroid cancer surgery,^[42]^ and middle ear surgery.^[39]^ Total intravenous anesthesia was used in 6 studies,^[29,31,35,40,43,47]^ whereas intravenous-inhalation combined anesthesia was used in 10 studies.^[33,34,38,39,42,44–46,48,49]^ Dexamethasone was used in 3 studies,^[33–35]^ and the antiemetics included ondansetron,^[34]^ palonosetron,^[35]^ tropisetron,^[29,31,33,38,40,43–46,48,49]^ dolasetron,^[42]^ azasetron,^[40]^ and granisetron.^[47]^ Penehyclidine was used during the induction of anesthesia,^[29,31,33,34,39,42,44–49]^ at the beginning of the operation,^[40]^ and at the end of surgery.^[35,38,43]^ Antiemetics were used during the induction of anesthesia,^[39,42]^ at the beginning of the operation,^[40]^ and at the end of the surgery.^[29,31,33–35,38,43–49]^

### 3.3. Bias risk assessment

**Figure 2.**
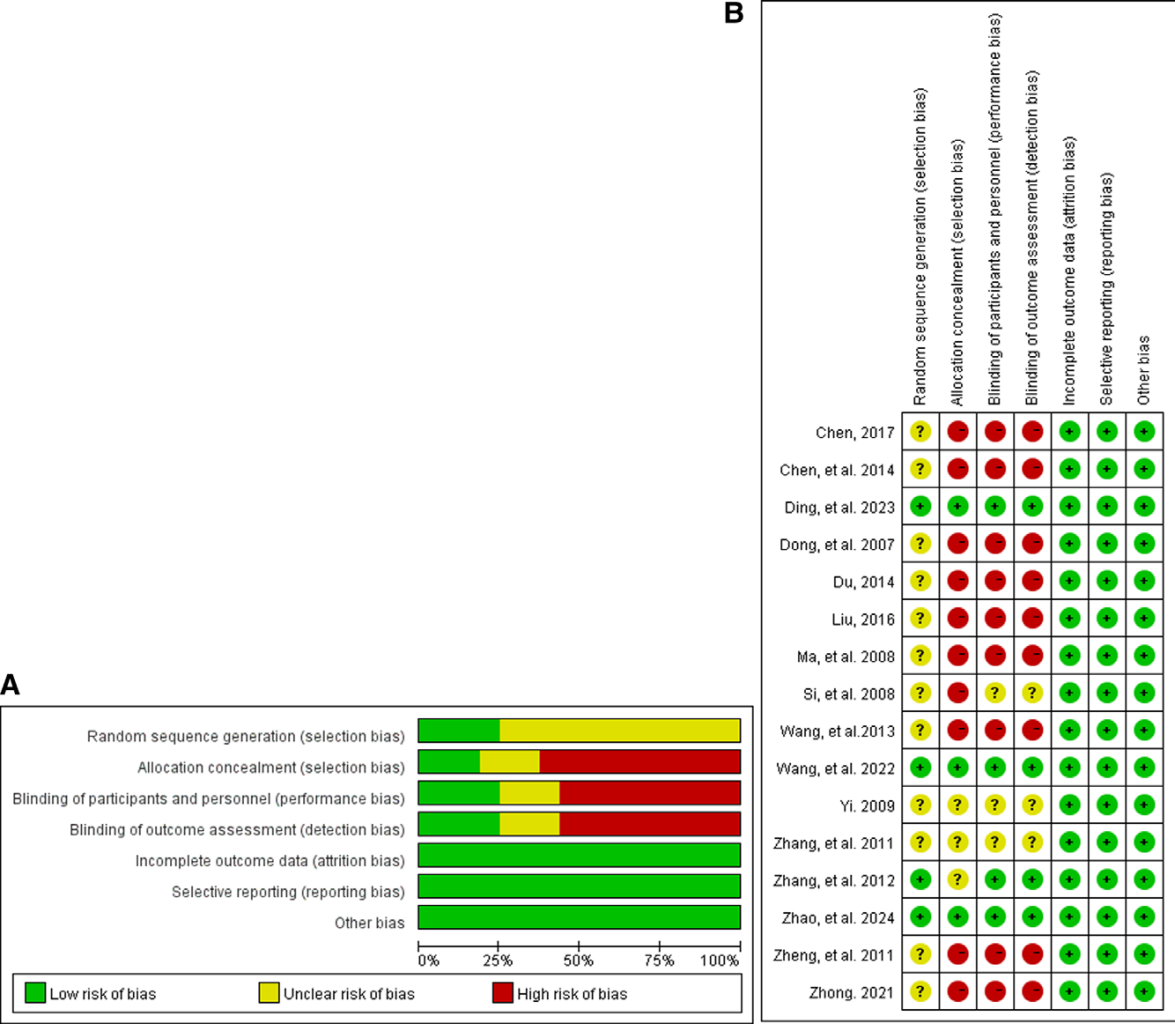
(A) Risk of bias assessment results for individual studies. (B) Summary plot for risk of bias assessment for individual studies.

Five of the included studies described the method of random sequence generation,^[29,33–35,42]^ 4 included studies described the blinding of participants and the blinding of outcome assessment,^[29,33–35]^ and only 3 included studies described allocation concealment.^[33–35]^ All included studies were confirmed as having a low risk of incomplete outcome data, reporting and other biases. The data demonstrated that our meta-analysis articles are of low quality (Fig. 2).

### 3.4. Outcomes

#### 3.4.1. Primary outcome: Incidence of PONV

**Figure 3.**
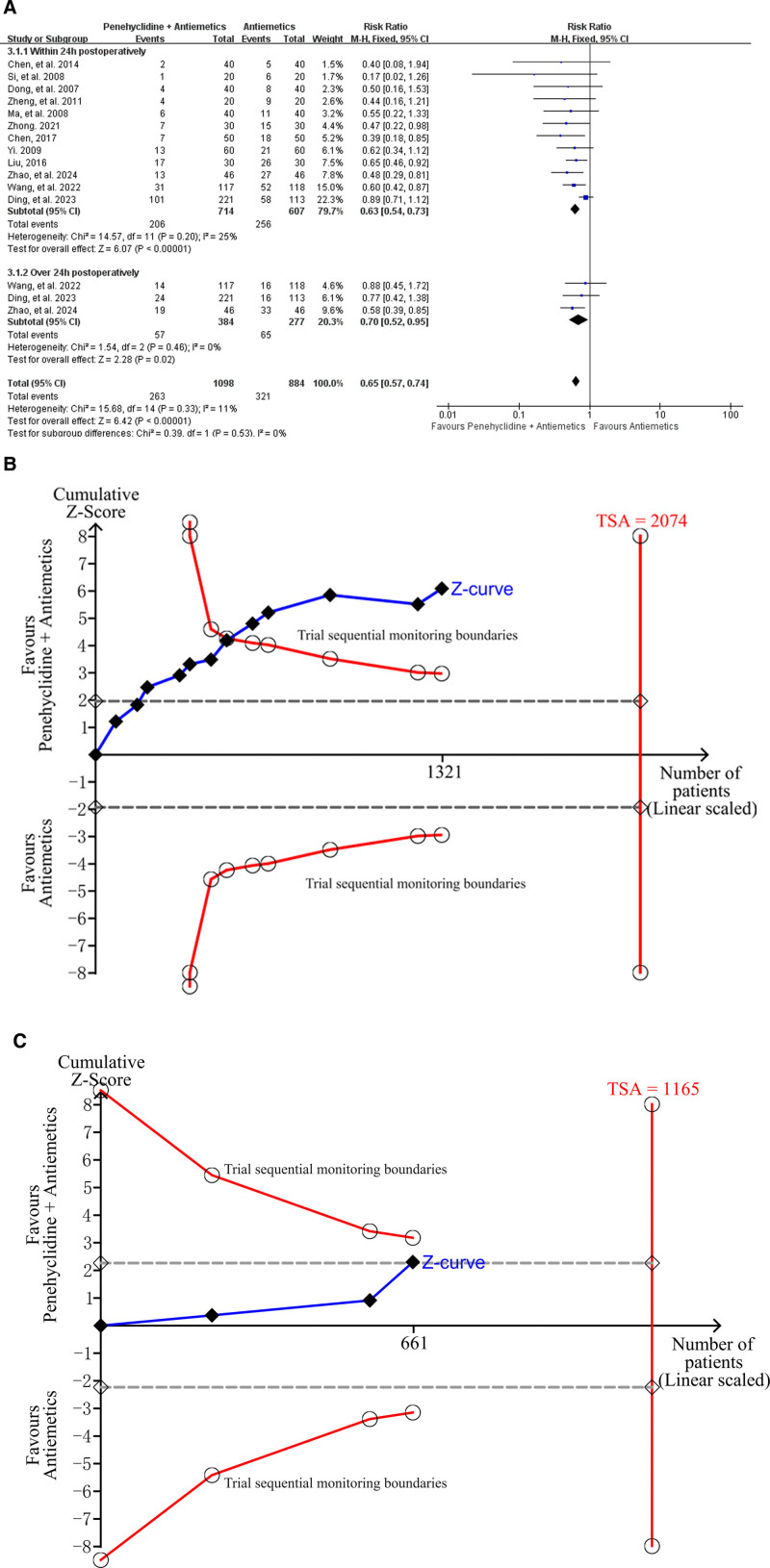
Forest plots and trial sequential analysis (TSA) of the incidence of postoperative nausea and vomiting (PONV). (A) Forest plot for the incidence of PONV. (B) TSA of the incidence of PONV within 24 h postoperatively: α = 2.5%; β = 90%; RR = 30%. The cumulative *Z*-curve crossed the trial sequential monitoring boundary, establishing sufficient and conclusive evidence. (C) TSA of the incidence of PONV over 24 h postoperatively: α = 2.5%; β = 90%; RR = 30%. The cumulative *Z*-curve did not cross the trial sequential monitoring boundary, establishing insufficient and uncertain evidence. The continuous blue full line filled with a Black square represents the cumulative *Z*-curve, the red full line with a blank circle represents the trial sequential monitoring boundaries, and the RIS. The gray dashed lines represent the conventional CIs. CI = confidence interval, PONV = postoperative nausea and vomiting, RIS = required information size, TSA = trial sequential analysis.

The incidence of PONV was reported in 12 studies,^[3–40,42,43,46–49]^ including 714 patients in the Penehyclidine + Antiemetics group and 607 in the Antiemetics group. Meta-analysis revealed that penehyclidine + Antiemetics group provided a lower incidence of PONV compared to Antiemetics group (23.95% vs 36.31%; RR = 0.65; 95% CI: 0.57–0.74; *P* < .00001; low heterogeneity; *I*^2^ = 11%, *P* = .33l; Fig. 3A). Subgroup analysis revealed that penehyclidine combined with antiemetics resulted in a lower incidence of PONV within 24 hours postoperatively (28.85% vs 42.17%; RR = 0.63, 95% CI: 0.54–0.73; *P* < .00001; low heterogeneity; *I*^2^ = 25%, *P* = .20; Fig. 3A) and over 24 hours postoperatively (14.84% vs 23.47%; RR = 0.70, 95% CI: 0.52–0.95; *P* = .02; low heterogeneity; *I*^2^ = 0%, *P* = .46; Fig. 3A). The test for subgroup differences indicated that, compared with the Antiemetics group, the Penehyclidine + Antiemetics group had a stable effect on reducing the incidence of PONV, and this effect was not affected by the varying postoperative periods (*P* = .53).

For the subgroup analysis of the incidence of PONV within 24 hours postoperatively, the TSA revealed that the accrued information size (n = 1321) reached 64% of the estimated RIS (n = 2074). The cumulative *Z* score crossed the trial sequential monitoring boundary (Fig. 3B). Therefore, the TSA of the pooled meta-analysis provided firm evidence for the anticipated intervention effect.

For the subgroup analysis of the incidence of PONV over 24 hours postoperatively, the TSA revealed that the accrued information size (n = 661) reached 57% of the estimated RIS (n = 2074). The cumulative *Z* score did not cross the trial sequential monitoring boundary (Fig. 3C). Therefore, the TSA of the pooled meta-analysis revealed uncertain evidence for the anticipated intervention effect, and further trials are needed.

#### 3.4.2. Secondary outcomes

##### 3.4.2.1. Incidence of different severities of PONV

**Figure 4.**
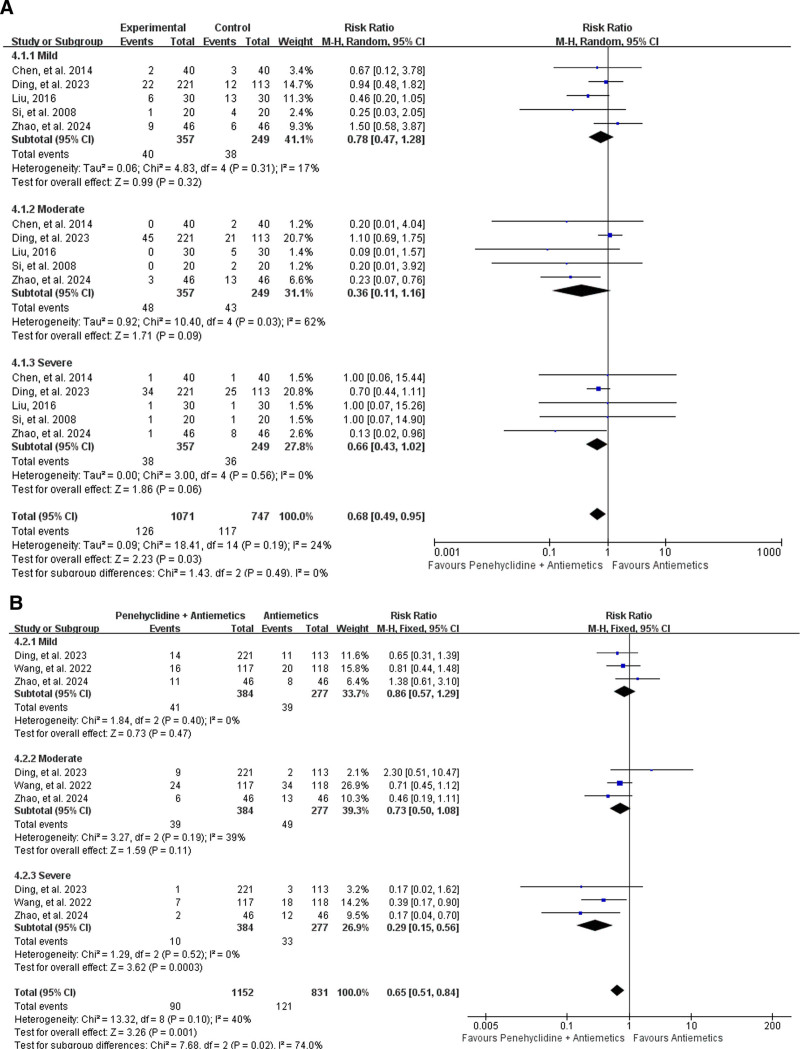
Forest plots for severity of PONV. (A) Forest plot for the incidence of PONV within 24 h postoperatively. (B) Forest plot for the incidence of PONV over 24 h postoperatively. PONV = postoperative nausea and vomiting.

Incidence of different severities of PONV within 24 hours postoperatively was reported in 5 studies,^[34,35,38–40]^ including 357 patients in the Penehyclidine + Antiemetics group and 249 in the Antiemetics group. Subgroup analysis revealed that there was no difference in the incidence of mild, moderate or severe PONV within 24 hours after surgery between the Penehyclidine + Antiemetics group and the Antiemetics group. For mild PONV (11.20% vs 15.26%; RR = 0.78, 95% CI: 0.47–1.28; *P* = .32; low heterogeneity, *I*^2^ = 17%, *P* = .31); for moderate PONV (13.44% vs 17.27%; RR = 0.36, 95% CI: 0.11–1.16; *P* = .09; high heterogeneity, *I*^2^ = 62%, *P* = .03); and for severe PONV (10.64% vs 14.56%; RR = 0.66, 95% CI: 0.43–1.02; *P* = .06; low heterogeneity, *I*^2^ = 0%, *P* = .56; Fig. 4A).

The incidence of different severities of PONV over 24 hours postoperatively was reported in 3 studies,^[33–35]^ including 384 patients in the Penehyclidine + Antiemetics group and 277 in the Antiemetics group. Subgroup analysis revealed that there was no difference between the Penehyclidine + Antiemetics group and the Antiemetics group in terms of the incidence of mild and moderate PONV over 24 hours postoperatively. For mild PONV (10.68% vs 14.08%; RR = 0.86, 95% CI: 0.57–1.29; *P* = .47; low heterogeneity, *I*^2^ = 0%; *P* = .40), for moderate PONV (10.16% vs 17.69%; RR = 0.73, 95% CI: 0.50–1.08; *P* = .11; low heterogeneity, *I*^2^ = 39%, *P* = .19; Fig. 4B). Pooled analysis revealed that penehyclidine combined with antiemetics was associated with a lower incidence of the severe PONV over 24 hours postoperatively (2.60% vs 11.91%; RR = 0.29, 95% CI: 0.15–0.56; *P* = .0003, low heterogeneity, *I*^2^ = 0%, *P* = .52; Fig. 4B).

##### 3.4.2.2. Incidence of postoperative rescue antiemetic therapy

**Figure 5.**
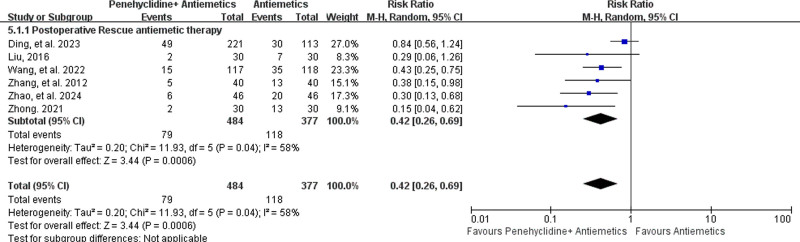
Forest plots for the incidence of postoperative rescue antiemetic therapy.

Postoperative rescue antiemetic therapy was reported in 6 studies,^[29,33–35,38,42]^ including 484 patients in the Penehyclidine + Antiemetics group and 377 in the Antiemetics group. Pooled analysis revealed that penehyclidine combined with antiemetics was associated with a lower incidence of postoperative rescue antiemetic therapy (16.32% vs 31.30%; RR = 0.42, 95% CI: 0.26–0.69; *P* = .0006; high heterogeneity, *I*^2^ = 58%, *P* = .04; Fig. 5).

##### 3.4.2.3. Incidence of postoperative complications

###### 3.4.2.3.1. Dry mouth

**Figure 6.**
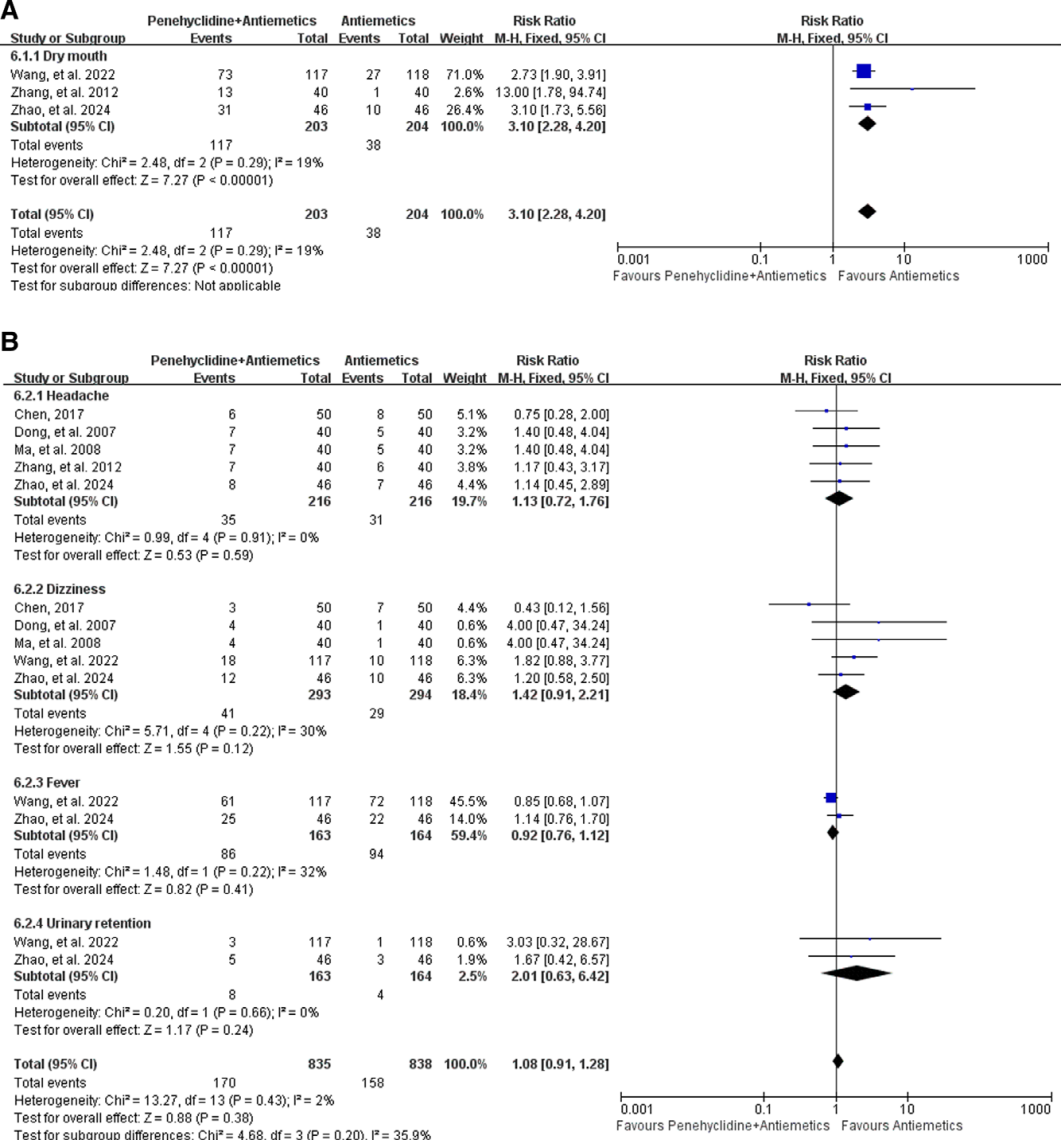
Forest plots for the incidence of postoperative complications. (A) Forest plot for the incidence of postoperative dry mouth. (B) Forest plot for the incidence of other postoperative complications.

Postoperative dry mouth was reported in 3 studies,^[29,33,34]^ including 203 patients in the Penehyclidine + Antiemetics group and 204 in the Antiemetics group. Pooled analysis revealed that penehyclidine combined with antiemetics was associated with a higher incidence of postoperative dry mouth (57.64% vs 18.63%; RR = 3.10, 95% CI: 2.28–4.20; *P* < .00001; low heterogeneity, *I*^2^ = 19%, *P* = .29; Fig. 6A).

###### 3.4.2.3.2. Other anticholinergic-related complications

Other anticholinergic-related complications (including headache, dizziness, fever, and urinary retention) were reported in 6 studies,^[29,33,34,43,48,49]^ including 333 patients in the Penehyclidine + Antiemetics group and 334 in the Antiemetics group. Pooled analysis revealed that there was no difference in the incidence of other anticholinergic-related complications between the Penehyclidine + Antiemetics group and Antiemetics group (20.36% vs 18.85%; RR = 1.08, 95% CI: 0.91–1.28; *P* = .38; low heterogeneity, *I*^2^ = 2%, *P* = .43; Fig. 6B).

Subgroup analysis revealed that there was no difference in the incidence of postoperative headache (16.20% vs 14.35%; RR = 1.13, 95% CI: 0.72–1.76; *P* = .59; low heterogeneity, *I*^2^ = 0%, *P* = .91; Fig. 6B), dizziness (13.99% vs 9.86%; RR = 1.42, 95% CI: 0.91–2.24; *P* = .12; low heterogeneity, *I*^2^ = 30%, *P* = .22; Fig. 6B), fever (52.76% vs 57.32%; RR = 0.92, 95% CI: 0.76–1.12; *P* = .41; low heterogeneity, *I*^2^ = 32%, *P* = .22; Fig. 6B), or urinary retention (4.91% vs 2.44%; RR = 2.01, 95% CI: 0.63–6.42; *P* = .24; low heterogeneity, *I*^2^ = 0%, *P* = .66; Fig. 6B).

#### 3.4.3. Exploratory outcomes

##### 3.4.3.1. Incidence of postoperative nausea (PON)

**Figure 7.**
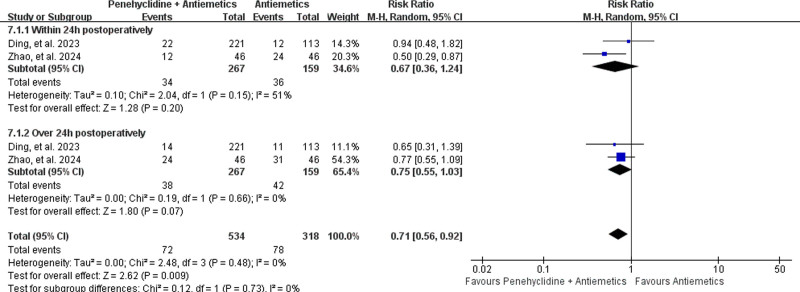
Forest plots for the incidence of postoperative nausea (PON). PON = postoperative nausea.

The incidence of PON was reported in 2 studies,^[34,35]^ including 267 patients in the Penehyclidine + Antiemetics group and 159 in the Antiemetics group. Meta-analysis revealed that the combination of penicyclidine with antiemetics provided a lower incidence of PON than did antiemetics alone (13.48% vs 24.53%; RR = 0.71, 95% CI: 0.56–0.92; *P* = .009; low heterogeneity, *I*^2^ = 0%, *P* = .48; Fig. 7).

Subgroup analysis revealed that there was no difference in the incidence of PON within 24 hours postoperatively (12.73% vs 22.64%; RR = 0.67, 95% CI: 0.36–1.24; *P* = .20; low heterogeneity, *I*^2^ = 0%, *P* = .15; Fig. 7) or over 24 hours postoperatively (14.23% vs 26.42%; RR = 0.75, 95% CI: 0.55–1.03; *P* = .07; low heterogeneity, *I*^2^ = 0%, *P* = .66; Fig. 7).

##### 3.4.3.2. Incidence of postoperative vomiting (POV)

**Figure 8.**
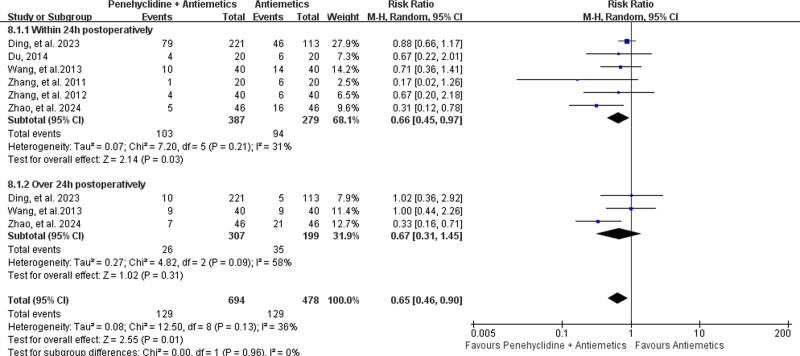
Forest plots for the incidence of postoperative vomiting (POV). POV = postoperative vomiting.

The incidence of POV was reported in 6 studies,^[29,31,34,35,44,45]^ including 387 patients in the Penehyclidine + Antiemetics group and 279 in the Antiemetics group. Meta-analysis revealed that penehyclidine combined with antiemetics resulted in a lower incidence of POV than did antiemetics alone (18.59% vs 26.99%; RR = 0.65, 95% CI: 0.46–0.90; *P* = .01; low heterogeneity, *I*^2^ = 36%, *P* = .13; Fig. 8).

Subgroup analysis revealed that penehyclidine combined with antiemetics was associated with a lower incidence of POV within 24 hours postoperatively (26.61% vs 33.69%; RR = 0.66, 95% CI: 0.45–0.97; *P* = .03; low heterogeneity, *I*^2^ = 31%, *P* = .21; Fig. 8). However, there was no difference in the incidence of POV over 24 hours postoperatively (8.47% vs 17.59%; RR = 0.67, 95% CI: 0.31–1.45; *P* = .31; high heterogeneity, *I*^2^ = 58%, *P* = .09; Fig. 8).

### 3.5. Risk of publication bias

**Figure 9.**
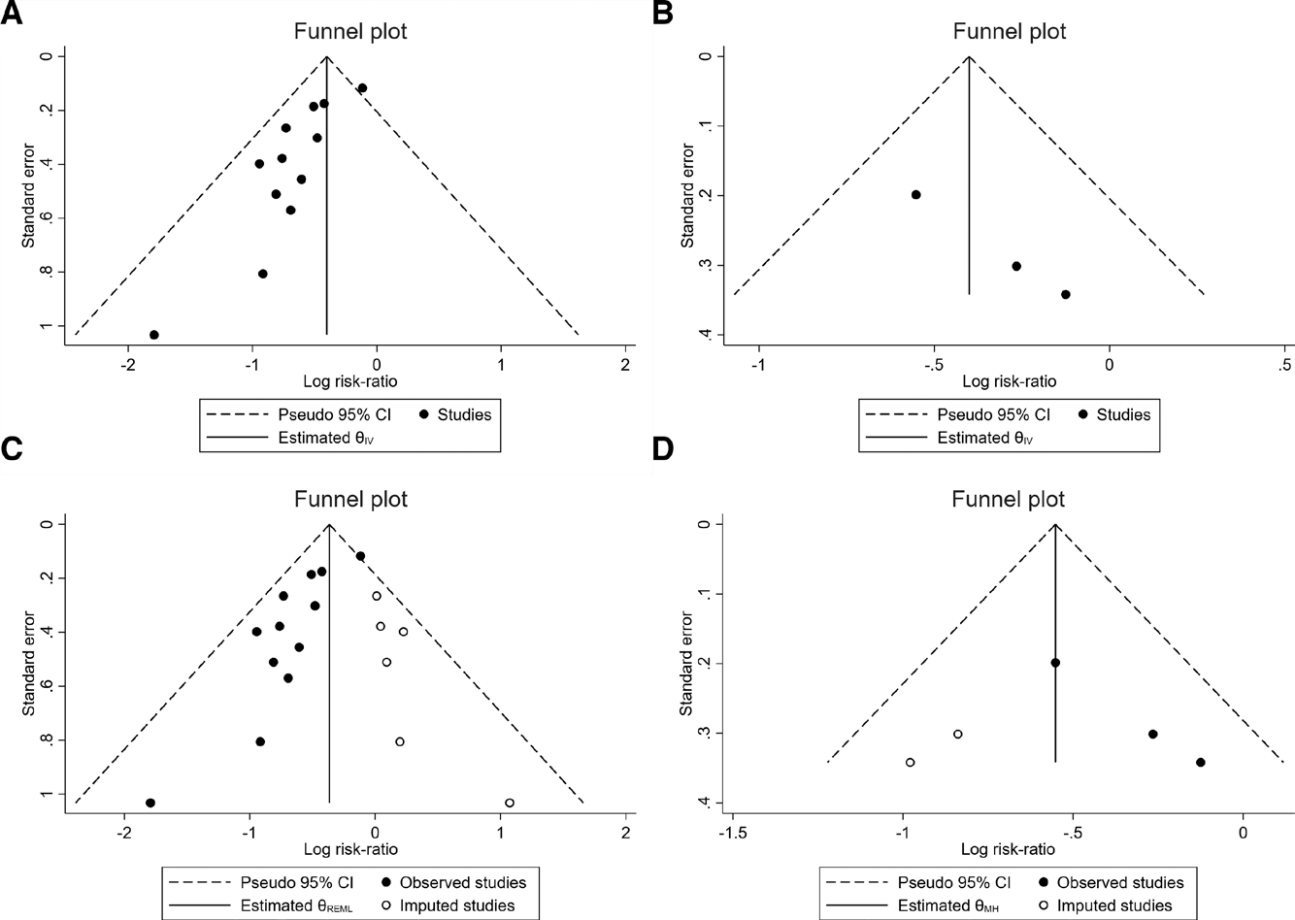
Funnel plot for publication bias of the studies included in the incidence of PONV. (A) Distribution of studies included in the incidence of PONV within 24 h postoperatively. (B) Distribution of studies included in the incidence of PONV over 24 h postoperatively. (C) Funnel plots of the incidence of PONV within 24 h postoperatively after applying the trim-and-fill method. The closed dots indicate the observed studies, and the white dots indicate the missing studies imputed by the trim-and-fill method. (D) Funnel plots of the incidence of PONV over 24 h postoperatively after applying the trim-and-fill method. The closed dots indicate the observed studies, and the white dots indicate the missing studies imputed by the trim-and-fill method. PONV = postoperative nausea and vomiting.

Significant publication bias was indicated among the included studies in both the incidence of PONV within 24 hours and over 24 hours postoperatively, as illustrated by the asymmetrical distribution of funnel plot tests (Fig. 9A, B) and Egger’s regression test (*P* = .0007 for the incidence of PONV within 24 hours postoperatively and *P* = .026 for the incidence of PONV over 24 hours postoperatively), respectively.

To reduce and adjust for publication bias in this study, trim-and-fill analysis was performed to estimate the number of missing studies that might exist.^[57]^ The original Log RR for PONV within 24 hours postoperatively was −0.51 (95% CI: −0.717–−0.303; Fig. 9A). After adding potentially 6 missing studies, the pooled Log RR was −0.364 (95% CI: −0.538 to −0.191; Fig. 9C). Egger’s regression test revealed that correction for potential publication bias did not materially alter the Log RR and the 95% CI of PONV within 24 hours postoperatively, implying that the result was stable. The original Log RR for PONV over 24 hours postoperatively was −0.40 (95% CI: −0.693 to −0.108; Fig. 9B). After adding potentially 2 missing studies, the pooled Log RR was −0.552 (95% CI: −0.796 to −0.308; Fig. 9D). Egger’s regression test revealed that correction for potential publication bias did not materially alter the Log RR and the 95% CI of PONV over 24 hours postoperatively, indicating that the result was stable.

### 3.6. Quality of evidence

We assessed the level of evidence of each outcome via the GRADE criteria, and the results are presented in Table 2. The evidence quality of the primary outcome was low and was downgraded by 2 levels. For the incidence of PONV within 24 hours postoperatively, the evidence quality was downgraded by 2 levels (the proportion of information from studies at high risk of bias is sufficient to affect the interpretation of results; and asymmetry in the funnel plot); For the incidence of PONV over 24 hours postoperatively, the evidence quality was downgraded by 2 levels (total number of events is <300; and <10 studies included). For secondary outcomes, the incidence of severe PONV over 24 hours postoperatively, dry mouth and other postoperative complications were low evidence quality and downgraded by 2 levels. However, the incidence of postoperative rescue antiemetic therapy was very low quality.

**Table 2:** The overall results of GRADE evaluation.

Outcomes		RR (95% CI)	No of participants (studies)	Quality of the evidence (GRADE)	Reasons
Incidence of PONV		RR 0.65 (0.57–0.74)	1982 (12 studies)	Low quality (⊕⊕⊝⊝)	1,2,3,8
Subgroup	Within 24 h postoperatively	RR 0.63 (0.54–0.73)	1321 (12 studies)	Low quality (⊕⊕⊝⊝)	1,2,3,8
	Over 24 h postoperatively	RR 0.7 (0.52–0.95)	661 (3 studies)	Low quality (⊕⊕⊝⊝)	4,5,6,8
Severity of PONV within 24 h postoperatively					
Subgroup	Mild	RR 0.78 (0.47–1.28)	606 (5 studies)	Low quality (⊕⊕⊝⊝)	4,5,6,8
	Moderate	RR 0.36 (0.11–1.16)	606 (5 studies)	Very low quality (⊕⊝⊝⊝)	4,5,6,7
	Severe	RR 0.66 (0.43–1.02)	606 (5 studies)	Low quality (⊕⊕⊝⊝)	4,5,6,8
Severity of PONV over 24 h postoperatively					
Subgroup	Mild	RR 0.86 (0.57–1.29)	606 (5 studies)	Low quality (⊕⊕⊝⊝)	4,5,6,8
	Moderate	RR 0.73 (0.5–1.08)	606 (5 studies)	Low quality (⊕⊕⊝⊝)	4,5,6,8
	Severe	RR 0.29 (0.15–0.56)	606 (5 studies)	Low quality (⊕⊕⊝⊝)	4,5,6,8
Postoperative Rescue antiemetic therapy		RR 0.42 (0.26–0.69)	861 (6 studies)	Very low quality (⊕⊝⊝⊝)	1,4,5,7
Complications					
Subgroup	Dry mouth	RR 3.1 (2.28–4.2)	407 (3 studies)	Low quality (⊕⊕⊝⊝)	4,5,6,8
	Other complications	RR 1.08 (0.91–1.28)	1673 (6 studies)	Low quality (⊕⊕⊝⊝)	1,2,5,8
Incidence of PON		RR 0.71 (0.56–0.92)	852 (2 studies)	Low quality (⊕⊕⊝⊝)	4,5,6,8
Subgroup	Within 24 h postoperatively	RR 0.67 (0.36–1.24)	426 (2 studies)	Very low quality (⊕⊝⊝⊝)	4,5,6,7
	Over 24 h postoperatively	RR 0.75 (0.55–1.03)	426 (2 studies)	Low quality (⊕⊕⊝⊝)	4,5,6,8
Incidence of POV		RR 0.65 (0.46–0.9)	1172 (6 studies)	Low quality (⊕⊕⊝⊝)	1,4,5,8
Subgroup	Within 24 h postoperatively	RR 0.66 (0.45–0.97)	666 (6 studies)	Low quality (⊕⊝⊝⊝)	1,4,5,8
	Over 24 h postoperatively	RR 0.67 (0.31–1.45)	506 (3 studies)	Very low quality (⊕⊝⊝⊝)	4,5,6,7

Reasons description: 1: The proportion of information from studies at high risk of bias is sufficient to affect the interpretation of results; 2: Total number of events is >300; 3: Asymmetry in funnel plot; 4: Total number of events is <300; 5: Less than 10 studies included; 6: Most information is from studies at low risk of bias; 7: Heterogenetiy > 50%; 8: Heterogenetiy < 50%.

CI = 95% confidence interval, GRADE = Grading of Recommendations Assessment, Development, and Evaluation, PON = postoperative nausea, PONV = postoperative nausea and vomiting, POV = postoperative vomiting, RR = rate ratio.

## 4. Discussion

Our meta-analysis demonstrated that penehyclidine combined with antiemetics provided a lower incidence of PONV within and over 24 hours postoperatively, lower incidence of severe PONV over 24 hours postoperatively, and a lower incidence of postoperative rescue antiemetic therapy. However, it was associated with a higher incidence of dry mouth, but did not increase the incidence of other anticholinergic-related complications.

Penehyclidine combined with antiemetics resulted in a lower incidence of PONV both within and over 24 hours postoperatively. We speculated that the following reasons may be responsible. First, penehyclidine may reduce the vagal reflex, which may inhibit vagal afferent activation by acting on M1-type receptors to mitigate PONV. Second, penehyclidine can relieve gastrointestinal smooth muscle spasms by acting on M3 cholinergic receptors in the digestive glands and smooth muscle of the digestive tract, which is effective in reducing postoperative gastric intraluminal pressure. Third, penehyclidine postconditioning could improve small intestinal mucosal injury and reduce damage to the barrier function of the small intestinal mucosa caused by limb ischemia-reperfusion.^[61]^ Accordingly, penehyclidine could improve the intestinal microcirculation, which is beneficial for preventing PONV. Fourth, penehyclidine can play an anti-inflammatory role by regulating inflammatory mediators, reducing the sensitivity of the vestibular system, and reducing the incidence of PONV.^[62,63]^

In addition, the elimination half-life of penehyclidine is approximately 24 hours, causing its preventive effect on PONV to diminish or disappear after 24 hours.^[29,30]^ While, Gan et al and Sah et al reported that combination therapy with scopolamine and 5-HT3 has a longer effective action time than monotherapy with 5-HT3 alone for preventing PONV.^[64,65]^ Similarly, Wang et al reported that tropisetron plus penehyclidine was more effective at preventing PONV within 72 hours after surgery than monotherapy with tropisetron.^[33]^ Our meta-analysis also revealed that penehyclidine combined with antiemetics was more effective at preventing PONV as it resulted in a lower incidence of severe PONV over 24 hours postoperatively than monotherapy with antiemetics. These findings could be explained by an additive or synergistic effect of the 2 drugs.^[66–68]^ Owing to the better efficacy of penehyclidine combined with antiemetics in preventing PONV, the incidence of postoperative rescue antiemetic therapy was lower (16.32% vs 31.30%; RR = 0.42; 95% CI: 0.26–0.69; *P* = .0006).

Dry mouth symptoms were observed in 117 individuals (57.64%) in the Penehyclidine + Antiemetics group, which was significantly higher than that in the Antiemetics group (38 individuals; 18.63%). This could be attributed to its impact on type 3 muscarinic acetylcholine receptors, leading to reduced glandular secretion from respiratory submucosal glands. As a novel anticholinergic and highly selective antagonist of both type 3 and type 1 muscarinic acetylcholine receptors, penehyclidine has fewer adverse effects than other anticholinergics.^[24]^ Then, penehyclidine is mainly used as a preanesthetic medication to reduce respiratory secretions without increasing the heart rate.^[25]^

No significant differences were found between the 2 groups regarding other anticholinergic-related side effects, such as headache, dizziness, fever or urinary retention. Delayed recovery of intestinal function and postoperative delirium (POD) are major concerns in the use of anticholinergic drugs.^[69–71]^ On the basis of the limited available data, we did not perform the meta-analysis for these 2 complications. Zhao et al reported that the median time for first flatus postoperatively was prolonged by 6.5 hours in patients who underwent gynecological laparoscopic surgery.^[34]^ However, Ding et al reported that the median time to first flatus postoperatively was prolonged by 1 hour in patients undergoing laparoscopic bariatric surgery.^[35]^ The perioperative administration of anticholinergic drugs is an important risk factor for POD.^[70,71]^ However, in a recent meta-analysis including 33 RCTs and 4017 patients, penehyclidine was not associated with increased the incidence of POD.^[72]^ In addition, Wang et al reported that penehyclidine did not increase the incidence of POD.^[33]^ Therefore, more RCTs are needed to investigate the adverse effects of penehyclidine in preventing PONV.

We also investigated the efficacy of penehyclidine combined with routine antiemetics in preventing PON and POV separately. Our meta-analysis revealed that, compared with monotherapy with antiemetics alone, penehyclidine combined with antiemetics provided a lower incidence of PON and POV. However, few trials have contributed data to the subgroup analysis of the incidence of PON and POV, indicating that subgroup analysis may not be able to detect the advantages of penehyclidine combined with antiemetics because of the insufficient sample size. Moreover, penehyclidine could play a role in preventing PONV by blocking both the type 1 and type 3 muscarinic acetylcholine receptors, which play important roles in the pathogenesis of PONV.^[22,27,28]^ Therefore, we still hypothesized that penehyclidine combined with antiemetics would reduce the incidence of PON and POV both within and over 24 hours postoperatively when more RCTs are included in each subgroup.

The risk factors for PONV included female sex, inhaled anesthesia for >60 minutes, high-risk procedures (cholecystectomy, laparoscopic, gynecologic), and age < 50 years.^[14]^ In addition, perioperative glucocorticoids have been used to reduce the incidence of PONV.^[73]^ In our meta-analysis, studies involving different types of surgery, different antiemetics, different administration times of penehyclidine and antiemetics, and different maintenance methods of anesthesia were included. Moreover, dexamethasone was used in 3 studies.^[33–35]^ Differences between the included studies could influence the quality evidence of the outcomes. However, owing to the insufficient available data from the included studies, subgroup analysis was not performed on these differences. Our results revealed low heterogeneity in the primary outcome (*P* = .33, *I*^2^ = 11%), and in the subgroup analysis of the primary outcome (within 24 hours postoperatively: *P* = .2, *I*^2^ = 25%, and over 24 hours postoperatively: *P* = .46, *I*^2^ = 0%). Hence, these differences among the included studies did not result in high heterogeneity, which indicated that the primary outcome was stable.

For the primary outcome, TSA was performed to test the validity, and publication bias was assessed to assess the stability. In addition, GRADE was performed to test the quality evidence of all the outcomes. The TSA of the pooled meta-analysis revealed strong evidence that penehyclidine combined with antiemetics provided a lower incidence of PONV within 24 hours postoperatively, and that evidence for the incidence of PONV over 24 hours postoperatively was uncertain. Significant publication bias was indicated, and trim-and-fill analysis was performed to reduce and adjust for publication bias. Fortunately, the correction for potential publication bias did not materially alter the stability of the results. The results of the GRADE analysis indicated that the evidence quality of the outcomes was low to very low, indicating that the clinical value of penehyclidine combined with antiemetics in preventing PONV is limited. Therefore, more RCTs are required in the future to improve the evidence quality of the results.

To the best of our knowledge, this is the first meta-analysis pooling and analyzing the efficacy and safety of penehyclidine combined with antiemetics for PONV. For the primary outcome, TSA was performed to test the validity, and publication bias was assessed by visual judgment of the funnel plot asymmetry, Egger’s regression test, and trim-and-fill analysis to check the stability of the primary outcome. In addition, GRADE was performed to test the evidence quality of all the outcomes.

There are also limitations to our analysis. First, the number of studies with eligible data was limited, and the sample size of each included study was small. Potential heterogeneity could exist, which could result in inadequate statistical power for the outcomes. Therefore, TSA was performed as an additional statistical analysis for the primary outcome. Second, potential clinical and methodological heterogeneity related to the patient population, type of surgery, anesthesia maintenance method, different antiemetics, and different administration times, which could influence the outcomes. Third, the pooled results were supported by a low or very low quality of evidence according to GRADE. Therefore, the clinical value of the results is limited. Therefore, future RCTs with larger sample sizes, clinical consistency, and methodological consistency are required to confirm our findings.

## 5. Conclusion

Based on current published evidence, compared with antiemetics, penehyclidine combined with antiemetics provides better prevention efficiency for PONV by decreasing the incidence of PONV (a lower incidence of PON and a lower incidence of POV) and severe PONV over 24 hours postoperatively. Penehyclidine combined with antiemetics is associated with a higher incidence of dry mouth, whereas the incidence of other anticholinergic-related complications is not different. These findings may provide a new prevention therapy for PONV. Further RCTs with larger sample sizes, consistent clinical assessments, and methodological consistency are required to improve our understanding of the subject.

## Author contributions

**Conceptualization:** Hongwei Zhang, Jianqiao Zheng, Lu Zhang, Li Du.

**Data curation:** Hongwei Zhang, Jianqiao Zheng, Lu Zhang.

**Formal analysis:** Hongwei Zhang, Jianqiao Zheng, Lu Zhang, Li Du.

**Methodology:** Jianqiao Zheng, Lu Zhang, Li Du.

**Software:** Li Du.

**Supervision:** Jianqiao Zheng, Lu Zhang, Li Du.

**Writing – original draft:** Hongwei Zhang, Jianqiao Zheng.

**Writing – review & editing:** Lu Zhang, Li Du.

## Supplementary Material


