# Supplementary material for: Penehyclidine combined with antiemetics for preventing postoperative nausea and vomiting: A meta-analysis of randomized control trials and trial sequential analysis
Source: Medicine (Baltimore). 2025 Jun 20;104(25):e42908. doi: 10.1097/MD.0000000000042908 (PMC12187308; doi:10.1097/MD.0000000000042908)
Supplement: Supplementary file 1 [file medi-104-e42908-s001.docx]

| Cochrane CENTRAL | Search Details |  |
| --- | --- | --- |
| #1 | (Postoperative Nausea and Vomiting):ti,ab,kw OR ("PONV"):ti,ab,kw (Word variations have been searched) | 16848 |
| #2 | (Penehyclidine):ti,ab,kw | 84 |
| #3 | #1 AND #2 | 19 |
| PUBMED | Search Details |  |
| #1 | "postoperative nausea and vomiting"[MeSH Terms] OR ("postoperative"[All Fields] AND "nausea"[All Fields] AND "vomiting"[All Fields]) OR "postoperative nausea and vomiting"[All Fields] OR ("postoperative nausea and vomiting"[MeSH Terms] OR ("postoperative"[All Fields] AND "nausea"[All Fields] AND "vomiting"[All Fields]) OR "postoperative nausea and vomiting"[All Fields] OR "ponv"[All Fields]) | 13,436 |
| #2 | "penehyclidine"[Supplementary Concept] OR "penehyclidine"[All Fields] | 156 |
| #3 | #1 AND #2 | 8 |
| Ovid MEDLINE | Search Details |  |
| 1 | (Postoperative Nausea and Vomiting).mp. [mp=title, book title, abstract, original title, name of substance word, subject heading word, floating sub-heading word, keyword heading word, organism supplementary concept word, protocol supplementary concept word, rare disease supplementary concept word, unique identifier, synonyms, population supplementary concept word, anatomy supplementary concept word] | 6704 |
| 2 | PONV.mp. or exp "Postoperative Nausea and Vomiting"/ | 5503 |
| 3 | Penehyclidine.mp. | 135 |
| 4 | 1 or 2 | 6863 |
| 5 | 3 and 4 | 7 |
| Embase | Search Details |  |
| 1 | (Postoperative Nausea and Vomiting).mp. [mp=title, abstract, heading word, drug trade name, original title, device manufacturer, drug manufacturer, device trade name, keyword heading word, floating subheading word, candidate term word] | 14561 |
| 2 | PONV.mp. or exp "postoperative nausea and vomiting"/ | 15307 |
| 3 | Penehyclidine.mp. or phencyclidine/ | 8429 |
| 4 | 1 or 2 | 16588 |
| 5 | 3 and 4 | 14 |
| Web of science | Search Details |  |
| 1 | (((((TS=(Postoperative Nausea and Vomiting)) OR TS=(PONV)) OR TI=(Postoperative Nausea and Vomiting)) OR AB=(Postoperative Nausea and Vomiting)) OR TI=(PONV)) OR AB=(PONV) and Preprint Citation Index (Exclude – Database) | 16296 |
| 2 | ((TS=(Penehyclidine)) OR TI=(Penehyclidine)) OR AB=(Penehyclidine) and Preprint Citation Index (Exclude – Database) | 255 |
| 3 | #2 AND #1 and Preprint Citation Index (Exclude – Database) | 9 |
| **Chinese Database** | Search Details |  |
| VIP | (((题名或关键词=盐酸戊乙奎醚 OR 题名或关键词=penehyclidine hydrochloride) OR 题名或关键词=长托宁) AND ((题名或关键词=恶心呕吐 OR (题名或关键词=nausea AND 题名或关键词=vomiting)) OR 题名或关键词=恶心和呕吐)) | 26 |
| CBM | ( "长托宁"[摘要:智能] OR "戊乙奎醚"[摘要:智能]) AND "恶心呕吐"[摘要:智能] AND "腔镜"[摘要:智能] | 22 |
| CNKI | （主题：恶心呕吐 + 恶心呕吐症状 + 恶心呕吐反应 + '恶心呕吐(ponv)' + 恶心呕吐防治 + 恶心呕吐评分;) AND（主题：盐酸戊乙奎醚 + '盐酸戊乙奎醚(长托宁)' + 盐酸戊乙奎醚注射液 + '盐酸戊乙奎醚(pche)' + '长托宁(盐酸戊乙奎醚)' + 小剂量盐酸戊乙奎醚） | 45 |
| Wanfang | ((主题=长托宁) OR 主题=盐酸戊乙奎醚) AND ((主题=腔镜) AND 主题=恶心呕吐) | 31 |
| **Clinical Trials** | **Search Details** |  |
| Clinicaltrials.gov | Intervention/treatment: Penehyclidine; Condition or disease: PONV \| postoperative nausea and vomiting | 3 |
| WHO ICTRP Trial registry | Postoperative nausea and vomiting AND Penehyclidine | 2 |
| Chinese Clinical Trials.gov | 注册题目：长托宁 | 4 |
|  | 注册题目：戊乙奎醚 | 21 |
